# 2-De­oxy-d-galactitol

**DOI:** 10.1107/S2414314626007947

**Published:** 2026-08-07

**Authors:** Tomoko Takashita, Yoji Horii, Akihide Yoshihara, Mizuki Aoe, Tomohiko Ishii

**Affiliations:** a Graduate School of Science for Creative Emergence Kagawa University, 2217-20 Hayashi-cho Takamatsu Kagawa 761-0396 Japan; b Department of Applied Biological Science Faculty of Agriculture Kagawa University, Miki Kagawa 761-0795 Japan; Katholieke Universiteit Leuven, Belgium

**Keywords:** crystal structure, hydrogen bonding, de­oxy sugar, de­oxy-galactitol

## Abstract

2-De­oxy-d-galactitol, C_6_H_14_O_5_, was prepared by reduction of 2-de­oxy-d-galactose with sodium borohydride (NaBH_4_) and crystallized from aqueous solution. Colorless block-shaped crystals suitable for single-crystal X-ray diffraction were obtained. The title compound crystallizes in the triclinic space group *P*1, with two crystallographically independent mol­ecules in the asymmetric unit. In the crystal, the mol­ecules are linked by O—H⋯O hydrogen bonds, forming a three-dimensional hydrogen-bonded network.

## Abstract 2

The title compound, 2-de­oxy-d-galactitol, was prepared by sodium borohydride reduction of 2-de­oxy-d-galactose and crystallized from water. Its crystal structure contains two crystallographically independent mol­ecules in the asymmetric unit, which are linked by O—H⋯O hydrogen bonds into a three-dimensional network.

## Graphical abstract

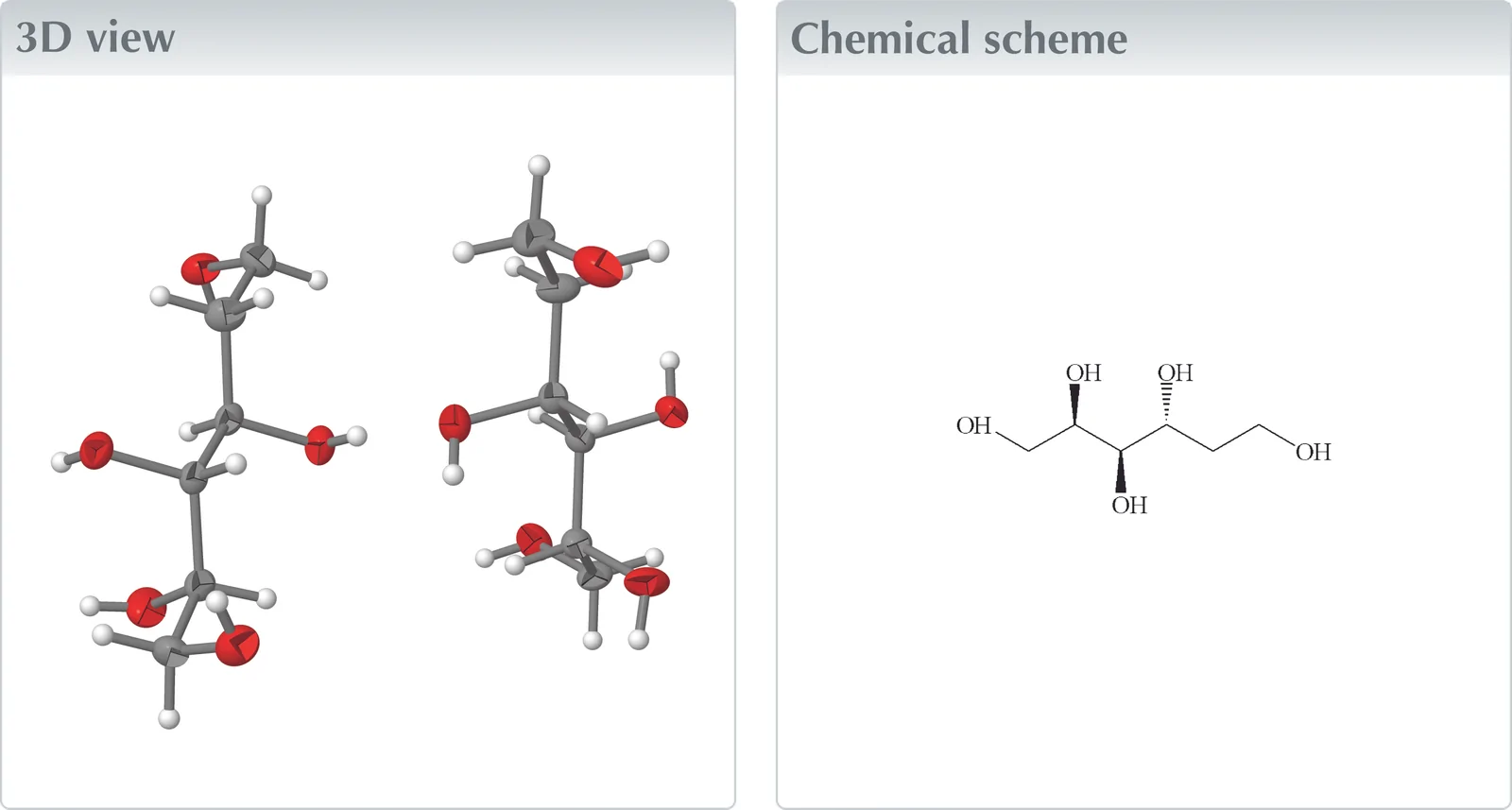

## Structure description

De­oxy sugars are carbohydrates in which one or more hy­droxy groups are replaced by hydrogen atoms. They occur in biological systems as components of physiologically important compounds; for example, 2-de­oxy-d-ribose forms part of the structural backbone of DNA (Nuevo *et al.*, 2018 ▸). De­oxy sugars have also attracted considerable inter­est because of their potential applications in pharmaceutical and agricultural fields. For example, 2-de­oxy-d-glucose and its analogues have been investigated as diagnostic and therapeutic agents (Pajak *et al.*, 2020 ▸), whereas 7-de­oxy-sedoheptulose has been investigated as a natural herbicidal agent (Brilisauer *et al.*, 2019 ▸). The title compound, C_6_H_14_O_5_, is a sugar alcohol obtained by reduction of the aldehyde group of 2-de­oxy-d-galactose. In the present study, single crystals of 2-de­oxy-d-galactitol were prepared to determine its crystal structure and characterize its inter­molecular inter­actions.

**Figure 1:**
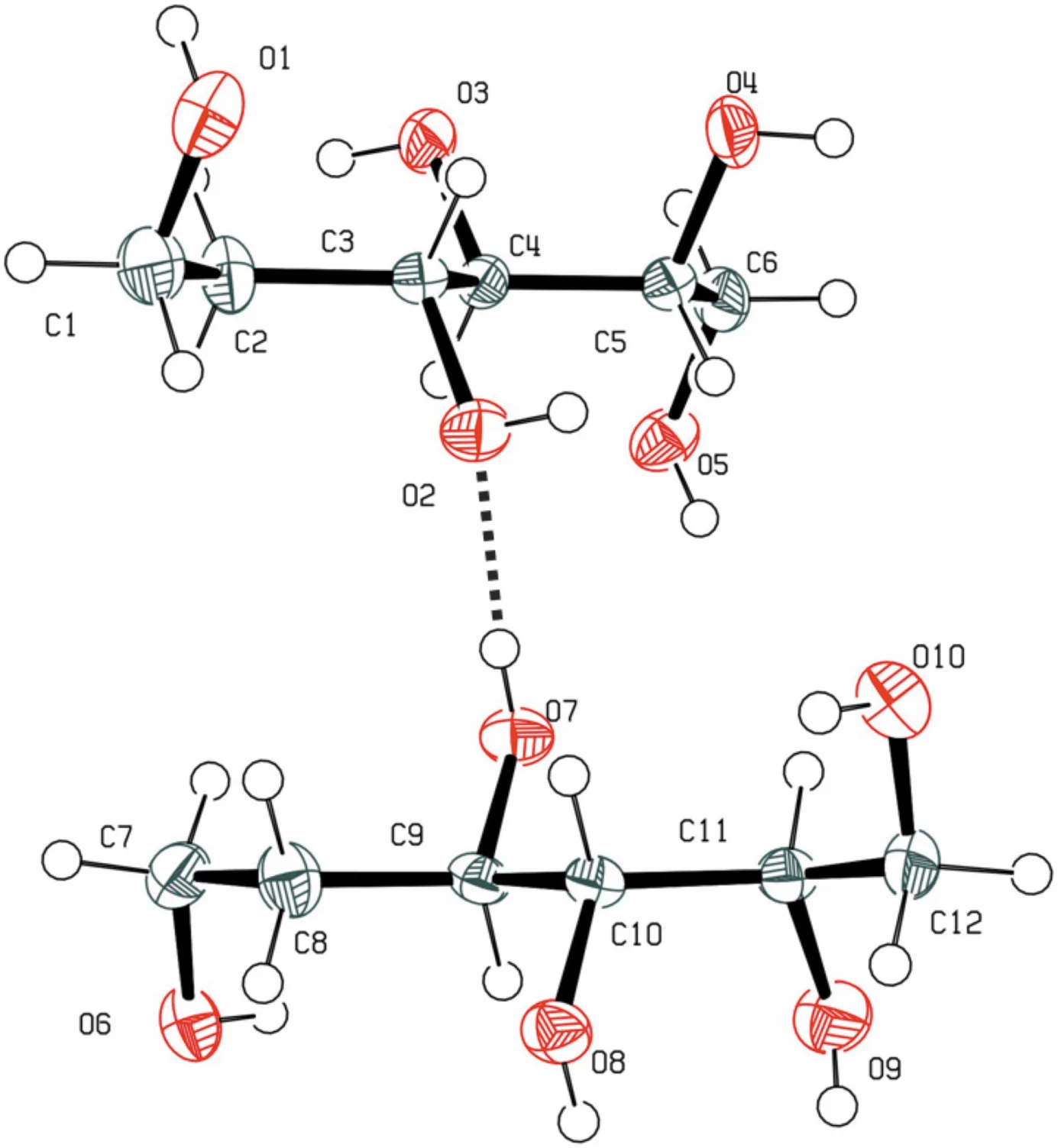
The title compound, showing the atom-labeling scheme. Displacement ellipsoids are drawn at the 50% probability level, and hydrogen atoms are shown as spheres of arbitrary radius. The O7—H7⋯O2 hydrogen bond is shown as a dashed line.

The title compound crystallizes in the triclinic space group *P*1. The asymmetric unit contains two crystallographically independent mol­ecules (Fig. 1 ▸) of 2-de­oxy-d-galactitol, which adopt very similar conformations, with an r.m.s. deviation of 0.530 Å after least-squares fitting. The stereogenic centres at C3, C4 and C5 in one molecule and at C9, C10 and C11 in the other molecule all have *S* configurations.

**Figure 2:**
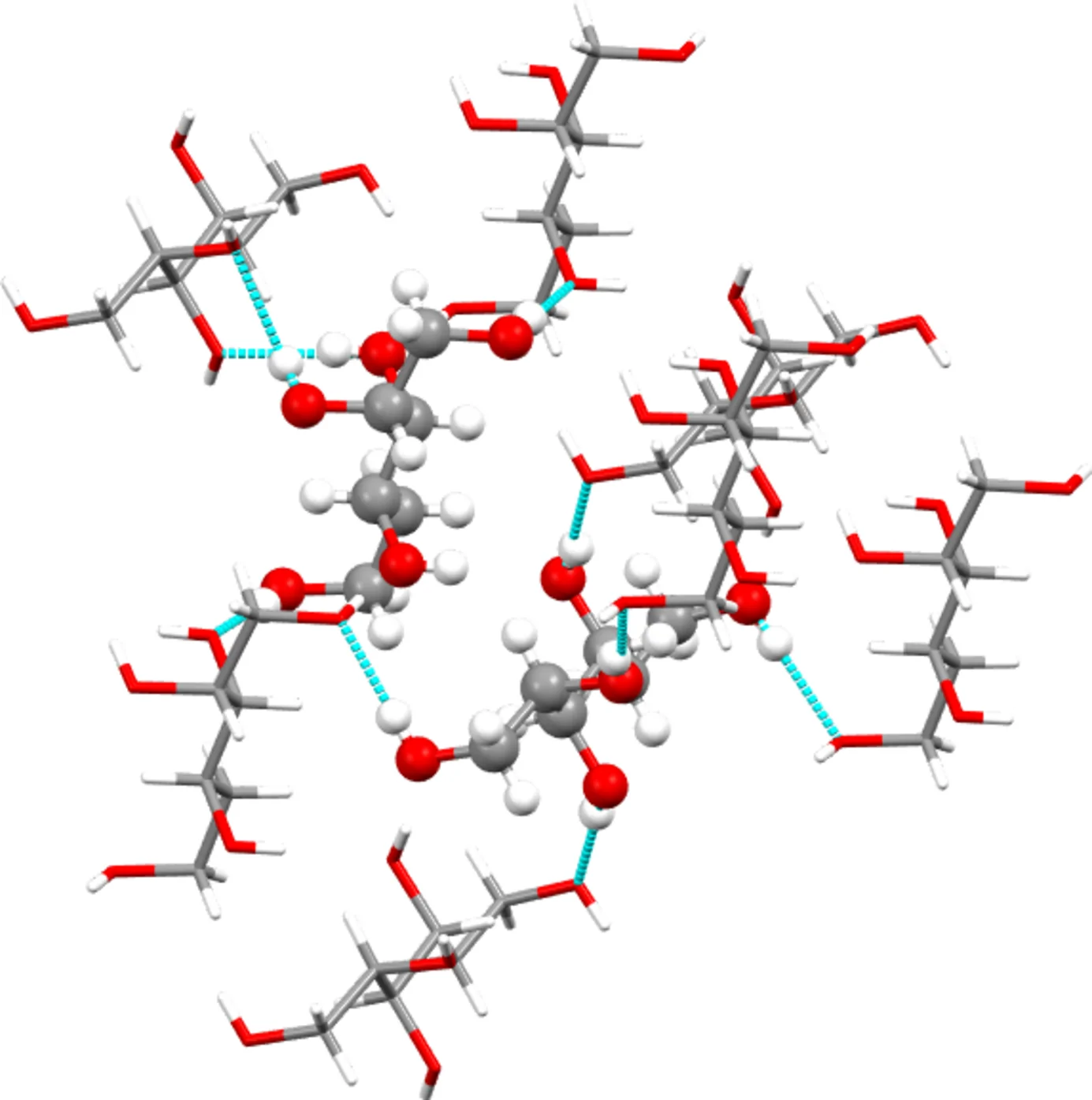
Partial crystal packing of the title compound, showing the nine crystallographically distinct O—H⋯O hydrogen bonds listed in Table 1 ▸. The two crystallographically independent mol­ecules in the asymmetric unit are shown in a ball-and-stick representation, and the surrounding symmetry-related mol­ecules are shown in a stick representation. Hydrogen bonds are shown as cyan dashed lines.

In the crystal, the mol­ecules are linked by the O—H⋯O hydrogen bonds listed in Table 1 ▸, with O⋯O distances ranging from 2.669 (4) to 2.872 (4) Å. These inter­actions connect the two crystallographically independent mol­ecules to surrounding symmetry-related mol­ecules, generating a three-dimensional hydrogen-bonded network. For clarity, the molecule containing atoms C1–C6/O1–O5 is designated *A*, and that containing atoms C7–C12/O6–O10 is designated *B*. The hydrogen-bonded network comprises two *A*⋯*A*, two *B*⋯*B* and five *A*⋯*B* hydrogen bonds. A partial packing diagram showing the nine crystallographically distinct hydrogen bonds is presented in Fig. 2 ▸.

## Synthesis and crystallization

2-De­oxy-d-galactitol was prepared by reduction of 2-de­oxy-d-galactose with sodium borohydride (NaBH_4_). Following purification, the product was dissolved in water, and the solution was allowed to evaporate slowly at room temperature. Colorless block-shaped single crystals suitable for single-crystal X-ray diffraction analysis were obtained.

## Refinement

Crystal data, data collection and structure refinement details are summarized in Table 2 ▸. The absolute configuration was assigned on the basis of the known configuration of the 2-de­oxy-d-galactose starting material and the synthetic route.

## Supplementary Material

Crystal structure: contains datablock(s) I. DOI: 10.1107/S2414314626007947/vm4078sup1.cif

Structure factors: contains datablock(s) I. DOI: 10.1107/S2414314626007947/vm4078Isup2.hkl

MS-Word file of Response to the Editor. DOI: 10.1107/S2414314626007947/vm4078sup3.docx

Supporting information file. DOI: 10.1107/S2414314626007947/vm4078Isup4.cml

CCDC reference: 2574888

Additional supporting information:  crystallographic information; 3D view; checkCIF report

## Figures and Tables

**Table 1:** Hydrogen-bond geometry (Å, °)

*D*—H⋯*A*	*D*—H	H⋯*A*	*D*⋯*A*	*D*—H⋯*A*
O1—H1⋯O6^i^	0.82	1.99	2.809 (4)	175
O2—H2⋯O5^ii^	0.82	1.85	2.669 (4)	172
O3—H3⋯O1^iii^	0.82	1.87	2.683 (4)	172
O4—H4⋯O6^iv^	0.82	2.06	2.871 (4)	168
O5—H5⋯O10^iii^	0.82	1.88	2.697 (4)	173
O6—H6⋯O8^iii^	0.82	1.95	2.764 (4)	176
O7—H7⋯O2	0.82	1.90	2.717 (4)	174
O8—H8⋯O3^v^	0.82	1.95	2.752 (4)	168
O10—H10⋯O7^ii^	0.82	1.87	2.685 (4)	176

Symmetry codes: (i) 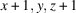; (ii) 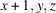; (iii) 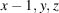; (iv) 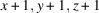; (v) 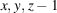.

**Table 2:** Experimental details

Crystal data	
Chemical formula	C_6_H_14_O_5_
*M*_r_	166.17
Crystal system, space group	Triclinic, *P*1
Temperature (K)	296
*a*, *b*, *c* (Å)	5.5249 (3), 8.0330 (4), 9.9771 (5)
α, β, γ (°)	108.091 (3), 107.597 (3), 93.072 (3)
*V* (Å^3^)	395.86 (4)
*Z*	2
Radiation type	Cu *K*α
μ (mm^−1^)	1.05
Crystal size (mm)	0.10 × 0.10 × 0.10

Data collection	
Diffractometer	Rigaku R-AXIS RAPID
Absorption correction	Multi-scan (*ABSCOR*; Rigaku, 1995 ▸)
*T*_min_, *T*_max_	0.666, 1.000
No. of measured, independent and observed [*I* > 2σ(*I*)] reflections	7108, 2577, 2115
*R*_int_	0.054
(sin θ/λ)_max_ (Å^−1^)	0.602

Refinement	
*R*[*F*^2^ > 2σ(*F*^2^)], *wR*(*F*^2^), *S*	0.040, 0.102, 1.11
No. of reflections	2577
No. of parameters	209
No. of restraints	3
H-atom treatment	H-atom parameters constrained
Δρ_max_, Δρ_min_ (e Å^−3^)	0.20, −0.26
Absolute structure	Flack *x* determined using 741 quotients [(*I*^+^)−(*I*^−^)]/[(*I*^+^)+(*I*^−^)] (Parsons *et al.*, 2013 ▸)
Absolute structure parameter	0.1 (3)

Computer programs: *RAPID-AUTO* (Rigaku, 2009 ▸), *SHELXT2018/2* (Sheldrick, 2015*a* ▸), *SHELXL2018/3* (Sheldrick, 2015*b* ▸) and *OLEX2* (Dolomanov *et al.*, 2009 ▸).

## full crystallographic data

### (2S,3S,4S)-Hexane-1,2,3,4,6-pentol Crystal data

**Table:**

C_6_H_14_O_5_	*Z* = 2
*M**_r_* = 166.17	*F*(000) = 180
Triclinic, *P*1	*D*_x_ = 1.394 Mg m^−^^3^
*a* = 5.5249 (3) Å	Cu *K*α radiation, λ = 1.54187 Å
*b* = 8.0330 (4) Å	Cell parameters from 4704 reflections
*c* = 9.9771 (5) Å	θ = 5.0–68.4°
α = 108.091 (3)°	µ = 1.05 mm^−^^1^
β = 107.597 (3)°	*T* = 296 K
γ = 93.072 (3)°	Block, clear light colourless
*V* = 395.86 (4) Å^3^	0.1 × 0.1 × 0.1 mm

### (2S,3S,4S)-Hexane-1,2,3,4,6-pentol Data collection

**Table:**

Rigaku R-AXIS RAPID diffractometer	2115 reflections with *I* > 2σ(*I*)
Detector resolution: 10.000 pixels mm^-1^	*R*_int_ = 0.054
ω scans	θ_max_ = 68.2°, θ_min_ = 5.0°
Absorption correction: multi-scan (ABSCOR; Rigaku, 1995)	*h* = −6→6
*T*_min_ = 0.666, *T*_max_ = 1.000	*k* = −9→9
7108 measured reflections	*l* = −12→11
2577 independent reflections	

### (2S,3S,4S)-Hexane-1,2,3,4,6-pentol Refinement

**Table:**

Refinement on *F*^2^	Hydrogen site location: inferred from neighbouring sites
Least-squares matrix: full	H-atom parameters constrained
*R*[*F*^2^ > 2σ(*F*^2^)] = 0.040	*w* = 1/[σ^2^(*F*_o_^2^) + (0.0458*P*)^2^] where *P* = (*F*_o_^2^ + 2*F*_c_^2^)/3
*wR*(*F*^2^) = 0.102	(Δ/σ)_max_ < 0.001
*S* = 1.11	Δρ_max_ = 0.20 e Å^−^^3^
2577 reflections	Δρ_min_ = −0.26 e Å^−^^3^
209 parameters	Absolute structure: Flack *x* determined using 741 quotients [(*I*^+^)-(*I*^-^)]/[(*I*^+^)+(*I*^-^)] (Parsons *et al.*, 2013)
3 restraints	Absolute structure parameter: 0.1 (3)
Primary atom site location: iterative	

### (2S,3S,4S)-Hexane-1,2,3,4,6-pentol Special details

**Table:**

Geometry. All esds (except the esd in the dihedral angle between two l.s. planes) are estimated using the full covariance matrix. The cell esds are taken into account individually in the estimation of esds in distances, angles and torsion angles; correlations between esds in cell parameters are only used when they are defined by crystal symmetry. An approximate (isotropic) treatment of cell esds is used for estimating esds involving l.s. planes.
Refinement. The structure was solved using SHELXT (Sheldrick, 2015*a*) and refined against F^2^ by full-matrix least-squares methods using *SHELXL* (Sheldrick, 2015*b*). All non-hydrogen atoms were refined anisotropically. Hydrogen atoms were placed in geometrically calculated positions and refined using constrained models. The final refinement gave *R*_1_ = 0.0398 for reflections with I > 2σ(I) and w*R*_2_ = 0.1021 for all data. The maximum and minimum residual electron densities were 0.20 and -0.26 e Å^-3^, respectively. The Flack parameter was 0.1 (3).

### (2S,3S,4S)-Hexane-1,2,3,4,6-pentol Fractional atomic coordinates and isotropic or equivalent isotropic displacement parameters (Å^2^)

**Table:**

	*x*	*y*	*z*	*U*_iso_*/*U*_eq_	
O1	0.9790 (6)	0.2113 (4)	0.7967 (3)	0.0344 (7)	
H1	0.951280	0.192463	0.867399	0.052*	
O2	0.6517 (6)	0.4837 (3)	0.6156 (3)	0.0286 (7)	
H2	0.763698	0.571124	0.647964	0.043*	
O3	0.3175 (6)	0.5092 (3)	0.8867 (3)	0.0280 (7)	
H3	0.219841	0.414283	0.853198	0.042*	
O4	0.6420 (5)	0.8103 (3)	0.9513 (3)	0.0325 (7)	
H4	0.697587	0.914177	0.968413	0.049*	
O5	−0.0103 (5)	0.7770 (3)	0.6970 (3)	0.0311 (7)	
H5	0.026231	0.800231	0.630335	0.047*	
C1	0.7495 (9)	0.1538 (6)	0.6698 (5)	0.0364 (12)	
H1A	0.694742	0.027849	0.644292	0.044*	
H1B	0.786037	0.170863	0.585392	0.044*	
C2	0.5335 (8)	0.2510 (5)	0.6953 (5)	0.0291 (10)	
H2A	0.487333	0.225510	0.774049	0.035*	
H2B	0.384511	0.206598	0.604636	0.035*	
C3	0.5985 (8)	0.4509 (5)	0.7382 (4)	0.0211 (8)	
H3A	0.752321	0.496450	0.827907	0.025*	
C4	0.3775 (8)	0.5449 (5)	0.7679 (4)	0.0201 (8)	
H4A	0.225600	0.499582	0.677063	0.024*	
C5	0.4361 (8)	0.7451 (5)	0.8122 (4)	0.0225 (9)	
H5A	0.490126	0.774013	0.736031	0.027*	
C6	0.2076 (8)	0.8350 (5)	0.8298 (4)	0.0269 (10)	
H6A	0.160662	0.811815	0.909253	0.032*	
H6B	0.257499	0.962298	0.859604	0.032*	
O6	−0.1063 (5)	0.1703 (4)	0.0491 (3)	0.0311 (7)	
H6	−0.165460	0.261660	0.075485	0.047*	
O7	0.3060 (5)	0.5254 (3)	0.3734 (3)	0.0281 (7)	
H7	0.412428	0.506920	0.442608	0.042*	
O8	0.6987 (5)	0.4824 (3)	0.1278 (3)	0.0267 (7)	
H8	0.597043	0.505391	0.059478	0.040*	
O9	0.4729 (5)	0.8068 (4)	0.2047 (3)	0.0367 (7)	
H9	0.515838	0.791988	0.130531	0.055*	
O10	1.1106 (6)	0.8219 (4)	0.4667 (3)	0.0331 (7)	
H10	1.175225	0.734351	0.436867	0.050*	
C7	0.0984 (9)	0.1598 (6)	0.1742 (5)	0.0311 (10)	
H7A	0.112195	0.036035	0.159814	0.037*	
H7B	0.055907	0.208740	0.264585	0.037*	
C8	0.3546 (8)	0.2575 (5)	0.1948 (5)	0.0296 (10)	
H8A	0.390655	0.213963	0.101756	0.035*	
H8B	0.487286	0.230472	0.270635	0.035*	
C9	0.3703 (8)	0.4578 (5)	0.2403 (4)	0.0222 (9)	
H9A	0.242377	0.483496	0.159939	0.027*	
C10	0.6339 (8)	0.5506 (5)	0.2608 (4)	0.0200 (8)	
H10A	0.760603	0.522465	0.340151	0.024*	
C11	0.6564 (8)	0.7525 (5)	0.3108 (4)	0.0243 (9)	
H11	0.617682	0.791801	0.404236	0.029*	
C12	0.9233 (8)	0.8456 (5)	0.3427 (4)	0.0290 (10)	
H12A	0.973244	0.800376	0.254567	0.035*	
H12B	0.919826	0.971421	0.362914	0.035*	

### (2S,3S,4S)-Hexane-1,2,3,4,6-pentol Atomic displacement parameters (Å^2^)

**Table:**

	*U*^11^	*U*^22^	*U*^33^	*U*^12^	*U*^13^	*U*^23^
O1	0.0300 (19)	0.0320 (16)	0.0498 (19)	0.0081 (14)	0.0169 (16)	0.0217 (14)
O2	0.034 (2)	0.0312 (16)	0.0245 (15)	0.0053 (14)	0.0139 (14)	0.0104 (12)
O3	0.0333 (19)	0.0246 (15)	0.0285 (16)	−0.0011 (13)	0.0132 (15)	0.0104 (12)
O4	0.0309 (17)	0.0198 (13)	0.0355 (15)	−0.0033 (12)	−0.0011 (13)	0.0074 (12)
O5	0.0250 (19)	0.0372 (17)	0.0351 (17)	0.0078 (14)	0.0076 (14)	0.0200 (14)
C1	0.046 (3)	0.027 (2)	0.035 (2)	0.012 (2)	0.015 (2)	0.0080 (19)
C2	0.026 (3)	0.019 (2)	0.037 (2)	0.0028 (18)	0.008 (2)	0.0059 (18)
C3	0.022 (2)	0.022 (2)	0.0177 (17)	0.0036 (18)	0.0045 (17)	0.0077 (15)
C4	0.020 (2)	0.0196 (19)	0.0208 (19)	0.0026 (17)	0.0061 (18)	0.0082 (15)
C5	0.025 (3)	0.021 (2)	0.0203 (19)	0.0042 (17)	0.0053 (18)	0.0078 (16)
C6	0.030 (3)	0.020 (2)	0.029 (2)	0.0103 (19)	0.010 (2)	0.0061 (17)
O6	0.0272 (19)	0.0254 (15)	0.0347 (15)	0.0009 (13)	0.0076 (14)	0.0053 (11)
O7	0.0285 (18)	0.0353 (17)	0.0230 (14)	0.0086 (14)	0.0112 (13)	0.0105 (13)
O8	0.031 (2)	0.0275 (14)	0.0233 (14)	0.0093 (13)	0.0121 (14)	0.0070 (12)
O9	0.043 (2)	0.0343 (15)	0.0356 (15)	0.0195 (14)	0.0111 (14)	0.0158 (13)
O10	0.031 (2)	0.0313 (16)	0.0318 (15)	0.0024 (14)	0.0086 (15)	0.0058 (12)
C7	0.034 (3)	0.029 (2)	0.032 (2)	0.003 (2)	0.010 (2)	0.0143 (19)
C8	0.023 (2)	0.027 (2)	0.034 (2)	0.0012 (19)	0.007 (2)	0.0073 (19)
C9	0.022 (2)	0.024 (2)	0.0158 (18)	0.0046 (17)	0.0030 (17)	0.0046 (15)
C10	0.019 (2)	0.0233 (19)	0.0165 (18)	0.0027 (17)	0.0049 (17)	0.0061 (15)
C11	0.031 (3)	0.022 (2)	0.0191 (19)	0.0061 (18)	0.0061 (18)	0.0074 (16)
C12	0.037 (3)	0.021 (2)	0.029 (2)	−0.002 (2)	0.012 (2)	0.0084 (18)

### (2S,3S,4S)-Hexane-1,2,3,4,6-pentol Geometric parameters (Å, º)

**Table:**

O1—H1	0.8200	O6—H6	0.8200
O1—C1	1.424 (5)	O6—C7	1.438 (5)
O2—H2	0.8200	O7—H7	0.8200
O2—C3	1.435 (4)	O7—C9	1.429 (4)
O3—H3	0.8200	O8—H8	0.8200
O3—C4	1.427 (4)	O8—C10	1.430 (4)
O4—H4	0.8200	O9—H9	0.8200
O4—C5	1.423 (5)	O9—C11	1.423 (4)
O5—H5	0.8200	O10—H10	0.8200
O5—C6	1.419 (4)	O10—C12	1.425 (5)
C1—H1A	0.9700	C7—H7A	0.9700
C1—H1B	0.9700	C7—H7B	0.9700
C1—C2	1.502 (6)	C7—C8	1.506 (6)
C2—H2A	0.9700	C8—H8A	0.9700
C2—H2B	0.9700	C8—H8B	0.9700
C2—C3	1.519 (5)	C8—C9	1.520 (5)
C3—H3A	0.9800	C9—H9A	0.9800
C3—C4	1.526 (4)	C9—C10	1.526 (4)
C4—H4A	0.9800	C10—H10A	0.9800
C4—C5	1.517 (5)	C10—C11	1.528 (5)
C5—H5A	0.9800	C11—H11	0.9800
C5—C6	1.517 (5)	C11—C12	1.511 (5)
C6—H6A	0.9700	C12—H12A	0.9700
C6—H6B	0.9700	C12—H12B	0.9700
			
C1—O1—H1	109.5	C7—O6—H6	109.5
C3—O2—H2	109.5	C9—O7—H7	109.5
C4—O3—H3	109.5	C10—O8—H8	109.5
C5—O4—H4	109.5	C11—O9—H9	109.5
C6—O5—H5	109.5	C12—O10—H10	109.5
O1—C1—H1A	109.0	O6—C7—H7A	109.0
O1—C1—H1B	109.0	O6—C7—H7B	109.0
O1—C1—C2	113.1 (4)	O6—C7—C8	113.0 (3)
H1A—C1—H1B	107.8	H7A—C7—H7B	107.8
C2—C1—H1A	109.0	C8—C7—H7A	109.0
C2—C1—H1B	109.0	C8—C7—H7B	109.0
C1—C2—H2A	108.8	C7—C8—H8A	108.7
C1—C2—H2B	108.8	C7—C8—H8B	108.7
C1—C2—C3	113.7 (4)	C7—C8—C9	114.2 (4)
H2A—C2—H2B	107.7	H8A—C8—H8B	107.6
C3—C2—H2A	108.8	C9—C8—H8A	108.7
C3—C2—H2B	108.8	C9—C8—H8B	108.7
O2—C3—C2	107.2 (3)	O7—C9—C8	110.2 (3)
O2—C3—H3A	109.4	O7—C9—H9A	107.8
O2—C3—C4	109.5 (3)	O7—C9—C10	110.8 (3)
C2—C3—H3A	109.4	C8—C9—H9A	107.8
C2—C3—C4	112.0 (3)	C8—C9—C10	112.2 (3)
C4—C3—H3A	109.4	C10—C9—H9A	107.8
O3—C4—C3	110.1 (3)	O8—C10—C9	110.8 (3)
O3—C4—H4A	108.7	O8—C10—H10A	107.2
O3—C4—C5	107.1 (3)	O8—C10—C11	111.0 (3)
C3—C4—H4A	108.7	C9—C10—H10A	107.2
C5—C4—C3	113.5 (3)	C9—C10—C11	113.1 (3)
C5—C4—H4A	108.7	C11—C10—H10A	107.2
O4—C5—C4	107.9 (3)	O9—C11—C10	111.5 (3)
O4—C5—H5A	109.1	O9—C11—H11	107.3
O4—C5—C6	108.7 (3)	O9—C11—C12	109.8 (3)
C4—C5—H5A	109.1	C10—C11—H11	107.3
C6—C5—C4	112.8 (3)	C12—C11—C10	113.3 (3)
C6—C5—H5A	109.1	C12—C11—H11	107.3
O5—C6—C5	113.2 (3)	O10—C12—C11	112.7 (3)
O5—C6—H6A	108.9	O10—C12—H12A	109.0
O5—C6—H6B	108.9	O10—C12—H12B	109.0
C5—C6—H6A	108.9	C11—C12—H12A	109.0
C5—C6—H6B	108.9	C11—C12—H12B	109.0
H6A—C6—H6B	107.8	H12A—C12—H12B	107.8
			
O1—C1—C2—C3	57.9 (5)	O6—C7—C8—C9	66.9 (5)
O2—C3—C4—O3	178.3 (3)	O7—C9—C10—O8	179.8 (3)
O2—C3—C4—C5	−61.7 (3)	O7—C9—C10—C11	−54.9 (3)
O3—C4—C5—O4	57.6 (4)	O8—C10—C11—O9	65.3 (4)
O3—C4—C5—C6	−62.6 (4)	O8—C10—C11—C12	−59.2 (4)
O4—C5—C6—O5	−179.2 (3)	O9—C11—C12—O10	169.6 (3)
C1—C2—C3—O2	61.7 (4)	C7—C8—C9—O7	55.7 (4)
C1—C2—C3—C4	−178.2 (3)	C7—C8—C9—C10	179.6 (3)
C2—C3—C4—O3	59.5 (3)	C8—C9—C10—O8	56.2 (4)
C2—C3—C4—C5	179.6 (3)	C8—C9—C10—C11	−178.4 (3)
C3—C4—C5—O4	−64.2 (3)	C9—C10—C11—O9	−60.0 (3)
C3—C4—C5—C6	175.7 (3)	C9—C10—C11—C12	175.6 (3)
C4—C5—C6—O5	−59.5 (4)	C10—C11—C12—O10	−65.0 (4)

### (2S,3S,4S)-Hexane-1,2,3,4,6-pentol Hydrogen-bond geometry (Å, º)

**Table:**

*D*—H···*A*	*D*—H	H···*A*	*D*···*A*	*D*—H···*A*
O1—H1···O6^i^	0.82	1.99	2.809 (4)	175
O2—H2···O5^ii^	0.82	1.85	2.669 (4)	172
O3—H3···O1^iii^	0.82	1.87	2.683 (4)	172
O4—H4···O6^iv^	0.82	2.06	2.871 (4)	168
O5—H5···O10^iii^	0.82	1.88	2.697 (4)	173
O6—H6···O8^iii^	0.82	1.95	2.764 (4)	176
O7—H7···O2	0.82	1.90	2.717 (4)	174
O8—H8···O3^v^	0.82	1.95	2.752 (4)	168
O10—H10···O7^ii^	0.82	1.87	2.685 (4)	176

Symmetry codes: (i) *x*+1, *y*, *z*+1; (ii) *x*+1, *y*, *z*; (iii) *x*−1, *y*, *z*; (iv) *x*+1, *y*+1, *z*+1; (v) *x*, *y*, *z*−1.
