# Supplementary material for: 2-De­oxy-d-galactitol
Source: IUCrdata. 2026 Aug 7;11(Pt 8):x260794. doi: 10.1107/S2414314626007947 (PMC13548379; doi:10.1107/S2414314626007947)
Supplement: Supplementary file 1 [file x-11-x260794-sup3.docx]

Response to Co-editor Professor L. Van Meervelt

Manuscript ID: vm4078

Title: 2-Deoxy-D-galactitol

We thank to Co-editor Professor L. Van Meervelt for the careful assessment of our manuscript and for the helpful comments. We have revised the manuscript and figures as described below.

**Comment 1:**

Line 25: add r.m.s. deviation of overlay of both molecules in the asymmetric unit.

**Response:**

The r.m.s. deviation for the overlay of the two crystallographically independent molecules has been added to the manuscript. Least-squares fitting in Olex2 gave an r.m.s. deviation of 0.530 Å without inversion. The revised sentence reads: “The two crystallographically independent molecules adopt very similar conformations, with an r.m.s. deviation of 0.530 Å after least-squares fitting.”

**Comment 2:**

Figure 1: missing – should be ortep-like drawing with atom labels.

**Response:**

Figure 1 has been added as an ORTEP-like representation showing the two crystallographically independent molecules in the asymmetric unit and the atom-labeling scheme. Displacement ellipsoids are drawn at the 50% probability level.

**Comment 3:**

Table 2: missing (H-bond geometry).

**Response:**

A hydrogen-bond geometry table has been added as Table 2. It lists all nine crystallographically distinct O—H···O hydrogen bonds, together with their geometric parameters and the corresponding symmetry codes.

**Comment 4:**

Figure 2: should also show all H-bonds listed in Table 2.

**Response:**

Figure 2 has been revised to show all nine O—H···O hydrogen bonds listed in Table 2. The two crystallographically independent molecules in the asymmetric unit are shown in a ball-and-stick representation, the surrounding symmetry-related molecules are shown in a stick representation, and the hydrogen bonds are represented by cyan dashed lines.

**Comment 5:**

References: some are not used in manuscript – check further and put references in alphabetical order!

**Response:**

The reference list has been carefully checked. References that were not cited in the manuscript or in the computer-program details have been removed, and the remaining references have been arranged in alphabetical order. The references for SHELXT and SHELXL have also been distinguished as Sheldrick (2015a) and Sheldrick (2015b), respectively.

Sincerely yours.

Tomohiko ISHII

Professor of Faculty of Engineering and Design, Kagawa University, Japan
